# Chemical Derivatization and Characterization of Novel Antitrypanosomals for African Trypanosomiasis

**DOI:** 10.3390/molecules26154488

**Published:** 2021-07-25

**Authors:** Aboagye Kwarteng Dofuor, Temitayo Samson Ademolue, Cynthia Mmalebna Amisigo, Kwaku Kyeremeh, Theresa Manful Gwira

**Affiliations:** 1 West African Center for Cell Biology of Infectious Pathogens, University of Ghana, Legon, Accra P.O. Box LG 54, Ghana; akdofuor@uesd.edu.gh (A.K.D.); tsademolue@gmail.com (T.S.A.); amismacyndyy@yahoo.com (C.M.A.); 2 Department of Biological, Physical and Mathematical Sciences, University of Environment and Sustainable Development, PMB, Somanya, Ghana; 3 Department of Biochemistry, Cell and Molecular Biology, University of Ghana, Legon, Accra P.O. Box LG 54, Ghana; 4 Department of Chemistry, University of Ghana, Legon, Accra P.O. Box LG 56, Ghana; kkyeremeh@ug.edu.gh

**Keywords:** *Trypanosoma brucei*, tortodofuordioxamide, tortodofuorpyramide, tortozanthoxylamide, *Z. zanthoxyloides*, antitrypanosomal

## Abstract

The search for novel antitrypanosomals and the investigation into their mode of action remain crucial due to the toxicity and resistance of commercially available antitrypanosomal drugs. In this study, two novel antitrypanosomals, tortodofuordioxamide (compound **2**) and tortodofuorpyramide (compound **3**), were chemically derived from the natural N-alkylamide tortozanthoxylamide (compound **1**) through structural modification. The chemical structures of these compounds were confirmed through spectrometric and spectroscopic analysis, and their in vitro efficacy and possible mechanisms of action were, subsequently, investigated in *Trypanosoma* *brucei* (*T. brucei*)*,* one of the causative species of African trypanosomiasis (AT). The novel compounds **2** and **3** displayed significant antitrypanosomal potencies in terms of half-maximal effective concentrations (EC_50_) and selectivity indices (SI) (compound **1**, EC_50_ = 7.3 μM, SI = 29.5; compound **2**, EC_50_ = 3.2 μM, SI = 91.3; compound **3**, EC_50_ = 4.5 μM, SI = 69.9). Microscopic analysis indicated that at the EC_50_ values, the compounds resulted in the coiling and clumping of parasite subpopulations without significantly affecting the normal ratio of nuclei to kinetoplasts. In contrast to the animal antitrypanosomal drug diminazene, compounds **1**, **2** and **3** exhibited antioxidant absorbance properties comparable to the standard antioxidant Trolox (Trolox, 0.11 A; diminazene, 0.50 A; compound **1**, 0.10 A; compound **2**, 0.09 A; compound **3**, 0.11 A). The analysis of growth kinetics suggested that the compounds exhibited a relatively gradual but consistent growth inhibition of *T. brucei* at different concentrations. The results suggest that further pharmacological optimization of compounds **2** and **3** may facilitate their development into novel AT chemotherapy.

## 1. Introduction

A major neglected tropical disease that is of significant health and economic concern to humans and livestock of Sub-Saharan Africa is African trypanosomiasis (AT), a tsetse-transmitted disease of humans and livestock caused by protozoan parasites of the *Trypanosoma* genus [1,2]. Even though the recent advances in the treatment of human AT are encouraging [3], the impact of animal AT on livestock productivity is still of great economic concern. Chemotherapy coupled with effective community screening remains the main mode of parasite control due to the absence of vaccines. However, the resistance and toxicity of commercially available drugs pose serious challenges to chemotherapy. Thus, there is the need to search for alternative sources of AT chemotherapy. 

The antitrypanosomal activities of several plants have been reported in different parts of the world [4,5,6,7]. We previously reported the antitrypanosomal activities of the Ghanaian plant species *Zanthoxylum zanthoxyloides* (Lam.) Zepern and Timler (*Z. zanthoxyloides*) in *Trypanosoma brucei* (*T. brucei*) [8]. The main secondary metabolites responsible for the antitrypanosomal properties of *Z. zanthoxyloides* were, subsequently, identified and characterized using various methods of spectrometry and spectroscopy [9,10]. Collectively, these are strong indications that natural plant products could serve as alternative sources of antitrypanosomal chemotherapy.

Despite their valuable medicinal values, natural products are usually beset with chemotherapeutic limitations that hinder their further development into drugs. These limitations include aspects of the potency, selectivity, toxicity, solubility, stability and bioavailability of compounds [11]. For instance, low chemical stability might have interfered with an efficient spectroscopic confirmation of lanyuamide, as well as the antitrypanosomal sensitivity of 9-oxo-10,12-octadecadienoic acid (9-oxo-ODA) upon their identification in *Z. zanthoxyloides*, as previously reported [9,10]. Moreover, the oxidant capacities of skimmianine and 9-oxo-ODA in *T. brucei* may serve as future limitations to their antitrypanosomal chemotherapeutic capacities as far as safety issues are concerned [10]. Thus, natural products usually require various forms of pharmacological optimization before their successful development into drugs can be achieved. 

N-alkylamides are a diverse group of bioactive natural plant products with reported pharmacological, nutritional, medicinal and cosmeceutical properties [12]. They consist of polyunsaturated fatty acids and relatively shorter aliphatic chains as well as a central amide bond at varying levels of cyclic and heteromolecular systems to the aliphatic moieties [12]. The potentially lipophilic properties of N-alkylamides could interfere with the identification of their targets due to the tendency to clump together instead of freely interacting with key metabolic proteins [12]. The aliphatic nature might also facilitate easy and uncontrolled access to the central nervous system, thereby ultimately maximizing the potentially toxic effects in the brain and spinal cord [13]. Thus, as with most natural products, N-alkylamides may require pharmacological improvements to be considered as promising chemotherapeutic agents.

We previously isolated and characterized a new antitrypanosomal N-alkylamide tortozanthoxylamide from *Z. zanthoxyloides* [9]. In order to improve the general pharmacological properties of tortozanthoxylamide, the present study sought to chemically derive and characterize two novel antitrypanosomals from the compound through structural modification. These derivatives were, subsequently, investigated in *T. brucei* for their potential in vitro efficacies and mechanisms of action. The results provide key insights into the antitrypanosomal capacity of the compounds for their development of chemotherapy for AT.

## 2. Results

### 2.1. Derivatization and Characterization of Compounds

We previously isolated and characterized the novel N-alkylamide antitrypanosomal N-(isobutyl)-3,4-methylenedioxy cinnamoyl amide (tortozanthoxylamide, compound **1**) from the root of the Ghanaian plant species *Z. zanthoxyloides* through bioactivity-guided chromatography, spectrometry and spectroscopy [9]. In order to improve the antitrypanosomal activities of compound **1**, we designed putative chemical derivatives of compound **1** based on the structural analysis of the available functionalities. The labile bond cleavage analysis of the mass fragmentation suggested the importance of the benzodioxole functionality in the base structure of compound **1** [9]. Pyrrolidine, a structure found in many natural alkaloids and synthetic drugs [14,15,16], was identified as a compact moiety for compound **1.** Coupled with the goal of reducing the aliphaticity of compound **1**, the 2-methylpropan-1-amine moiety or its 2-methylpropyl substructure was replaced by a pyrrolidine or benzodioxole functionality, respectively (Table 1). This led to the derivatization of 2-propenamide, N,3-bis(1,3-benzodioxol-5-yl)-(2E) (compound **2**) and 2-propen-1-one,3-(1,3-benzodioxol-5-yl)-1-(1-pyrrolidinyl)-(2E) (compound **3**) from the parent natural compound **1**. These derivatives were commercially synthesized by Aurora Fine Chemicals Limited, Graz, Austria (compound **2**, Chemical Abstracts Service (CAS) registry number = 1056639-88-3; compound **3**, CAS = 261913-19-3).

Compounds **2** and **3** were subjected to Gas chromatographic-mass spectrometric (GC-MS) analysis. The interpretation of mass spectra was conducted using a robust database of the National Institute of Standard and Technology (NIST). The mass fragmentation patterns of compounds **2** and **3** indicated an approximate *m*/*z* of 311 and 245, respectively (Supplementary Figures S2 and S4). The chromatographic elution of compounds in the GC-MS analysis occurred at retention times of 31.86 (compound **2**) and 21.80 (compound **3**) (Supplementary Figures S1 and S3). The IR spectra of both compounds displayed absorbance intensities within a range of wavenumbers suggestive of N-H (1650–1580 cm^−1^), C-C (1500–1400 cm^−1^) and C-N (1250–1020 cm^−1^) bends and stretches, as evident in the aromatic and amide groups of compounds **2** and **3** (Supplementary Figures S2 and S4). The UV–VIS spectra were restricted to absorption bands consistent with transitions of aromatic and amide moieties (250–350 nm) (Supplementary Figure S5). Chemical shifts from ^1^H and ^13^C NMR spectra reflective of shifting effects due to carbonyl (^1^H:10 ppm) and aromatic substitutions (^1^H: 6.5–7.5 ppm; ^13^C: 120–130 ppm) were evident in both compounds (Supplementary Figures S6 and S7). The 1D and 2D NMR data are summarized in Supplementary Tables S1 and S2. 

### 2.2. Antitrypanosomal Potency Analysis

The antitrypanosomal activities of compounds in *T. brucei* were determined in a 48-h alamar blue cell viability analysis. As shown in their dose–response curves, compounds **2** and **3** displayed potent antitrypanosomal activities in terms of their EC_50_ values (compound **1**, EC_50_ = 7.3 μM; compound **2**, EC_50_ = 3.2 μM; compound **3**, EC_50_ = 4.5 μM) (Table 2; Supplementary Figure S8). In the presence of normal mouse macrophages (RAW 264.7 cell lines), compounds **2** and **3** were more selective to *T. brucei* than compound **1**, as evidenced by their selectivity indices (SI) (compound **1**, SI = 29.5; compound **2**, SI = 91.3; compound **3**, SI = 69.9) (Table 2).

### 2.3. Antioxidant Capacity of Compounds in T. brucei

**Figure 1:**
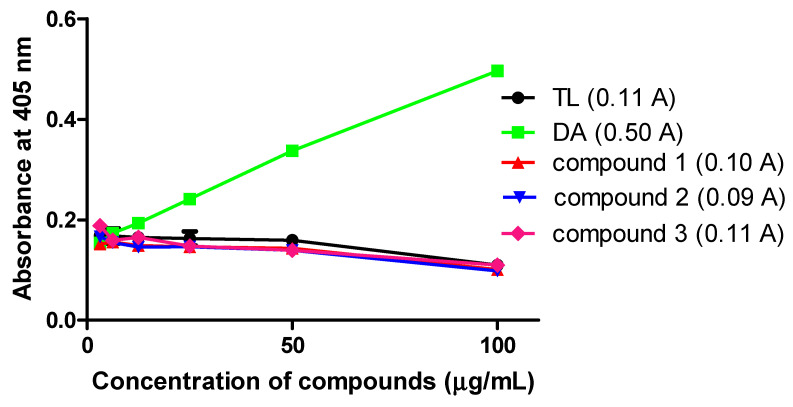
Antioxidant activities of compounds in *T. brucei*: Absorbance readings were recorded within a concentration range of 3.125 to 100 µg/mL. Antioxidant capacities were estimated from duplicate absorbance recordings at the maximum observed concentration of 100 µg/mL using the Trolox curve as the standard antioxidant. DA = Diminazene aceturate; TL = Trolox; A = absorbance units at 100 µg/mL.

The antioxidant properties of the compounds in *T. brucei* were determined by employing the reducing properties of ABTS (2,2′-azino-bis (3-ethylbenzthiazoline-6-sulfonic acid). For a standard antioxidant such as the water-soluble analog of vitamin E (Trolox), a dose-dependent reduction in the absorbance of the ABTS radical at 405 nm is expected [10]. Thus, a dose-dependent increase in the absorbance for a compound in *T. brucei* is an indication of oxidant activity. As was shown previously [10], the animal antitrypanosomal drug diminazene exhibited a strong oxidant potential (Figure 1). At the maximum tested concentration of 100 μg/mL, compounds **1**, **2** and **3** exhibited absorbance intensities that were not significantly different from Trolox, thereby suggesting antioxidant properties comparable to Trolox (Trolox, 0.11 A; compound **1**, 0.10 A; compound **2**, 0.09 A; compound **3**, 0.11 A) (Figure 1).

### 2.4. Antitrypanosomal Sensitivity Analysis of Compounds

**Figure 2:**
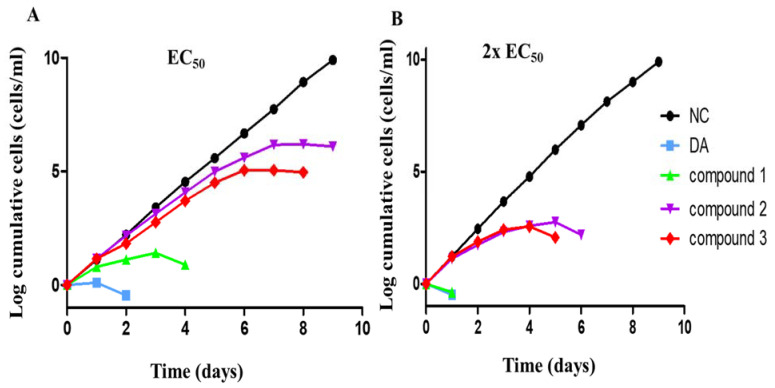
Compound sensitivity analysis in *T. brucei*: The growth kinetics of *T. brucei* was investigated after treatment with diminazene (DA), compounds **1**, **2** and **3** for 9 days at EC_50_ and 2 × EC_50_. NC = negative control.

The cumulative growth of parasites was monitored for 9 days at EC_50_ and 2 × EC_50_ values of compounds. As expected, there was no effect on the cumulative growth of *T. brucei* in the untreated cells (Figure 2). However, all the compounds resulted in a consistent growth inhibition of parasites with respect to time and dose (Figure 2). At the EC_50_ values, compounds **1**, **2** and **3** resulted in the complete eradication of parasites after 96, 192 and 216 h, respectively (Figure 2A). At 2 × EC_50_ values, both the diminazene- and compound **1**-treated cells were exterminated within 24 h, while all the compound **2**- and **3**-treated parasites were eliminated at a relatively slow rate, within 120 and 144 h, respectively (Figure 2B). 

### 2.5. Effects of Compounds on the Structure and Distribution of T. brucei

**Figure 3:**
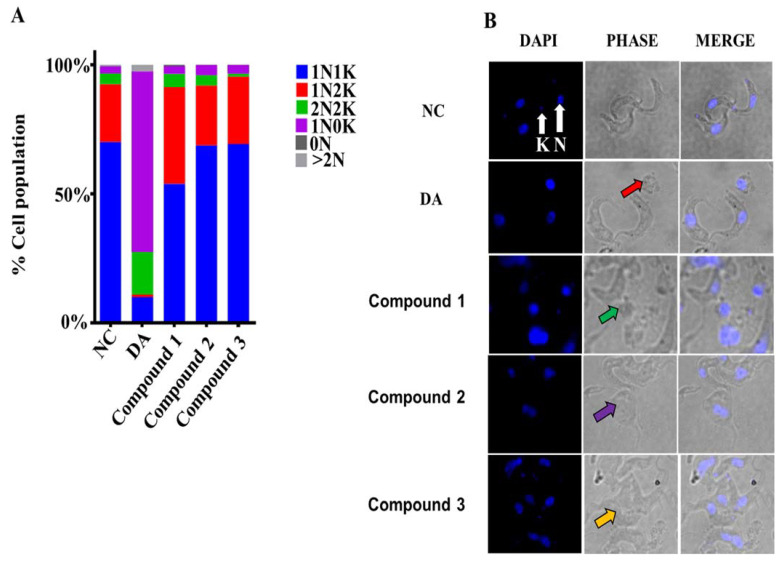
Effects of compounds on structure and distribution of *T. brucei: T. brucei* cells were treated at the EC_50_ values of compounds. (**A**) For each compound, percentage cell population was calculated from an average count of 240 cells in 10 microscopic fields. (**B**) Red arrow = DA-treated cells; green arrow = compound **1**-treated subpopulation; purple arrow = compound **2**-treated subpopulation; orange arrow = compound **3**-treated subpopulation; K = kinetoplast; N = nucleus; PHASE = Phase contrast; DAPI = 4′,6-diamidino-2-phenylindole; DA = Diminazene aceturate; NC = Negative control.

The effects of compounds on the morphology and distribution of *T. brucei* were investigated using fluorescence microscopy. The elongated slender shape of *T. brucei*, helical flagella and normal ratio of nuclei to kinetoplasts under untreated conditions (1N1K, 1N2K and 2N2K) were observed in the negative control (Figure 3A,B; Table S3). At the EC_50_ values, diminazene caused the loss of kinetoplasts in about 70% of the cells, in contrast to the approximately 3, 4 and 3% of cells for compounds **1**, **2** and **3**, respectively (Figure 3A; Table S3). However, amongst selected subpopulations, the compounds induced significant cell clumping and coiling of the normal spiral shape of parasites at the tested EC_50_ values (Figure 3B). 

## 3. Discussion

The natural products of plants are endowed with several pharmacological and medicinal properties. However, they are usually beset with pharmacological limitations that hinder their utilization or subsequent development into drugs. With the goal of dealing with this challenge, the antitrypanosomal tortozanthoxylamide (compound **1**) was chemically modified into tortodofuordioxamide (compound **2**) and tortodofuorpyramide (compound **3**). The subsequent chemical and antitrypanosomal characterization demonstrates how insights into the structural and functional properties of plant antitrypanosomals could facilitate their modification into novel compounds for the purpose of drug discovery.

The chemical derivatization increased the period of time required for the complete eradication of parasites, despite a relative increase in selectivity and potency. This slow rate of action may also account for the insignificant impact on the ratio of nuclei to kinetoplasts, despite the corresponding deformation of parasite morphology at EC_50_ values. Even though this property may not be temporally advantageous in terms of the time-dependent action of the compounds, it could have spatial benefits with regard to the effects on a selected spectrum of parasite populations as well as a minimization of the potential toxicity on host cells. Future studies that would provide insights into reaction kinetics at longer time periods are, therefore, encouraged. 

The excessive production of reactive oxygen species may serve as a source of damage to cells. The mode of action of nifurtimox, a human African antitrypanosomal drug, is proposed to involve the release of free radical and non-radical forms of reactive oxygen species that could damage proteins, DNA and lipids [17]. The animal African antitrypanosomal drug, diminazene, is known to cause damage to the kidney, liver and brain [18], of which a high oxidant activity could play an essential role [10]. Furthermore, despite the promising efficacies of other natural antitrypanosomals similarly isolated from *Z. zanthoxyloides*, there is the possibility that their significant oxidant activities may have damaging effects on host cells [10]. Thus, the antioxidant properties of the compounds, exhibited through the possible inhibition of reactive oxygen species in the parasites, can be advantageous in the context of the tendency to reduce the overall toxic effects that arise from oxidative stress.

Moreover, a combined effect of antitrypanosomal and antioxidant properties could have pharmacological advantages. In one study, derivatives of 4-hydroxycoumarins were shown to be good antioxidants and moderate antitrypanosomals [19]. A selected series of synthetic hydroxy-3-arylcoumarins were also reported to exhibit varying levels of antioxidant and trypanocidal activities potentially beneficial to the control of Chagas disease [20]. In light of their reducing properties, several natural antioxidants have also been proposed as adjuvants or supplementary therapy for the treatment of Chagas disease [21]. This is due to the potential induction of *T. cruzi*-mediated oxidative stress in host cells, which aids the progression of Chagas disease [21]. Thus, the inhibition of oxidative stress in the parasites could be of importance as far as the mechanisms of action of compounds **1**, **2** and **3** are concerned. However, despite these chemotherapeutic advantages, natural antioxidants may also aid the establishment of trypanosomes, which calls for careful moderation in their usage [22]. 

Collectively, this study paves the way for a further pharmacological evaluation of tortodofuordioxamide and tortodofuorpyramide to facilitate their development into commercially available antitrypanosomal drugs. The advantageous chemotherapeutic properties of tortodofuordioxamide and tortodofuorpyramide against *T. brucei* included improved parasite selectivity indices, the preservation of antioxidant properties and consistency in growth inhibition. Future studies may seek to investigate ways of increasing the rate of growth inhibition while retaining or improving other beneficial properties. Future studies should also focus on identifying targets of the compounds in *T. brucei* to shed light on the mechanisms of antitrypanosomal sensitivities. 

## 4. Materials and Methods

### 4.1. Culture of Parasites and Mammalian Cell Lines

Blood stream forms of the subspecies *T. brucei brucei* (*T. b. brucei*) (GUTat 3.1 strains) were cultured in vitro to the logarithm phase using Hirumi’s Modified Iscove’s Medium (HMI9, Thermo Fisher Scientific, Oxford, UK) with 10% fetal bovine serum (Thermo Fisher Scientific) at 5% CO_2_ and 37 °C. Mouse macrophages (RAW 264.7 cell lines, Sigma-Aldrich, Kent, 91062702) were cultivated in vitro to the logarithm phase using Dulbecco‘s Modified Eagle Media (DMEM, Thermo Fisher Scientific, Oxford, UK) with 10% fetal bovine serum at 5% CO_2_ and 37 °C.

### 4.2. Derivatization, Spectrometric and Spectroscopic Analysis

The natural compound **1** previously isolated from *Z. zanthoxyloides* [9] was used as the template for the design of compounds **2** and **3**. We designed putative chemical derivatives of compound **1** based on the structural analysis of available functionalities. Labile bond cleavage analysis suggested the importance of benzodioxole functionality in the base structure of compound **1**. Pyrrolidine was also employed as another compact moiety in the design process. The 2-methylpropan-1-amine moiety or its 2-methylpropyl substructure was replaced by a pyrrolidine or benzodioxole functionality. This led to the derivatization of 2-propenamide, N,3-bis(1,3-benzodioxol-5-yl)-(2*E*) (compound **2**) and 2-propen-1-one,3-(1,3-benzodioxol-5-yl)-1-(1-pyrrolidinyl)-(2*E*) (compound **3**) from the parent natural compound **1**. These structural designs were submitted to Aurora Fine Chemicals Limited, Graz, Austria, for custom synthesis. Compounds **2** and **3** are commercially available at Aurora Fine Chemicals Limited with the following Chemical Abstracts Service (CAS) registry numbers (unique registered chemical identifiers): compound **2**, CAS = 1056639-88-3; compound **3**, CAS = 261913-19-3. The experimental protocols employed in the synthesis of compounds **2** and **3** are copyrighted to Aurora Fine Chemicals Limited, Graz, Austria. GC-MS analysis of compounds **2** and **3** was performed using a PerkinElmer GC Clarus 580 Gas Chromatograph interfaced to a Mass Spectrometer PerkinElmer (Clarus SQ 8S) equipped with Elite-5MS (5% diphenyl/95% dimethyl polysiloxane) fused to a capillary column (L × I.D. 30 m × 0.25 mm, df 0.25 µm). The oven temperature was programmed from 40 °C with a 3 °C/min increase to 90 °C, then 10 °C/min to 240 °C and holding for 15 min at 240 °C. For GC-MS detection, an electron ionization system was operated in electron impact mode with ionization energy of 70 eV. Helium gas (99.999%) was used as a carrier gas at a constant flow rate of 1 mL/min and injection volume of 1 µL. The injector temperature and ion-source temperature were 250 and 150 °C, respectively. Mass spectra were taken at 70 eV with a scan interval of 0.1 s and fragments from 45 to 450 Da. The solvent delay was 0 to 2 min with a total GC-MS running time of about 42 min. The mass-detector used in this analysis was a PerkinElmer TurboMass (Software = TurboMass version 6.1.0.). Interpretation of mass spectra was conducted using the database of the National Institute of Standard and Technology (NIST). Mid-infrared (IR) spectroscopy was performed using the Attenuated Total Reflectance (ATR) spectrometer with the following specifications: instrument model = BRUKER ALPHA FT-IR platinum ATR; software version = OPUS-7.2.139.1294; number of scans = 24. UV–VIS spectroscopy was performed with the SPECORD 200 PLUS-223E1451 designation using the following specifications: lamp change = 320 nm; measurement mode = spectral scan; range = 200–700 nm; delta lambda = 1 nm; speed = 50 nm/s. All NMR spectra were acquired with a Bruker FT-NMR Avance 500 spectrometer (Ettlingen, Germany) at 300 K. All solvents used were of the HPLC grade.

### 4.3. Analysis of Cell Viability and Cytotoxicity

The subspecies *T. b. brucei* were seeded at a density of 1.5 × 10^5^ cells/mL in 96-well plates in a two-fold dilution of compounds and incubated for 24 h. Normal mouse macrophages (RAW 264.7) were initially seeded at a density of 1.5 × 10^5^ cells/mL for 48 h to allow for sufficient adherence to plates before treatment with the compounds in a two-fold dilution and subsequent incubation for another 24 h. Resazurin (10% *v*/*v*) was added to wells and incubated for another 24 h. Experiments were run in quadruplicates. Spectrophotometric absorbance was recorded at a wavelength of 570 nm. Diminazene aceturate (Sigma-Aldrich, Kent, UK), a known antitrypanosomal drug, was used as a positive control. 

### 4.4. Antioxidant Analysis of Antitrypanosomals

The ABTS (2,2′-azino-bis (3-ethylbenzthiazoline-6-sulfonic acid) antioxidant assay kit (Sigma-Aldrich, Kent, UK) was used for the investigation of the antioxidant capacity of compounds by following the manufacturer’s protocols. We followed the same procedures as elaborated previously with minor modifications [10]. Briefly, *T. b. brucei* cells were seeded at a density of 1.5 × 10^5^ cells/mL on 96-well plates in a two-fold dilution of compounds. Myoglobin was added to each well and incubated for 24 h. ABTS was added to each well and incubated for approximately 5 min at room temperature. After inactivating the reaction by adding a stop solution, absorbance was read at 405 nm. The final volume of cells and reagents in each well was 200 µL. Trolox ((±)-6-hydroxy-2,5,7,8-tetramethylchromane-2-carboxylic acid) was used as the positive control antioxidant. Experiments were performed in duplicates. 

### 4.5. Growth Kinetics Analysis

*T. b. brucei* cells were grown to a density of 1 × 10^6^ cells/mL and split into fresh media with the antitrypanosomal compounds at an initial cell density of 1 × 10^5^ cells/mL (day 0). Cells were then monitored and subcultured every 24 h by treating with the compounds at 1 × 10^5^ cells/mL for the next 8 days.

### 4.6. Fluorescence Microscopy

*T. b. brucei* cells were treated with compounds at the EC_50_ values for 24 h and centrifuged at 27,000 rpm for 10 min. Cells were resuspended in 1 mL of FBS-free HMI9 media and incubated for 30 min. Cells were pelleted and resuspended in 1 mL of FBS-free HMI9 media and incubated for another 30 min. Fixation was performed by incubating cells at 4 °C for 1 h in 1 mL of 8% paraformaldehyde in Voorheis modified PBS. Washing of cells was carried out by pelleting and resuspending in PBS, after which 10–20 μL of cell suspension was spread on poly-L-lysine-coated microscope slides, sprayed and wiped clean with 70% ethanol. The slides were allowed to air-dry for 15 min in a humid chamber and placed in a container with methanol at −20 °C for 30 min. The slides were rinsed in PBS, after which 0.1 μg/mL DAPI was added to the cells. Slides were rinsed again in PBS and 30 μL of mounting media was applied, along with coverslips and sealed with nail varnish for observation with the Zeiss Axio Vert.A1 inverted microscope. Data were analyzed with Image J version 2.1.0/1.53c. 

### 4.7. Statistical Analysis

Data from the cell viability, cytotoxicity, antitrypanosomal sensitivity and antioxidant activity assays were analyzed with GraphPad Prism version 5 (Graph Pad Software, San Diego, CA, USA). The half-maximal effective concentration (EC_50_) was calculated as the concentration that caused a 50% reduction in cell viability. EC_50_ values were calculated from a non-linear regression model using the Hill function. *p*-values < 0.05 were considered to be significant.

## Figures and Tables

**Table 1:** Chemical structures and molecular weights of compounds and moieties.

Name	Molecular Weight (g/mol)	Chemical Structure
Tortozanthoxylamide (Compound **1**)	247.29	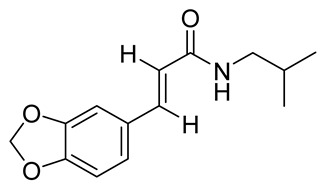
Tortodofuordioxamide (Compound **2**)	311.29	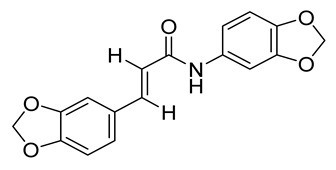
Tortodofuorpyramide (Compound **3**)	245.27	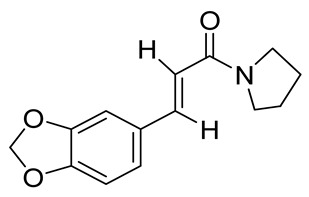
Benzodioxole	122.12	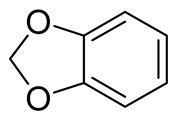
Pyrrolidine	71.12	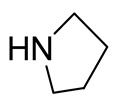

Compound **1** was used as the template for the design and synthesis based on structural insights in functionalities and labile bond cleavage analysis.

**Table 2:** Antitrypanosomal potencies and selectivity indices of compounds.

COMPOUNDS Mean EC_50_ ± SEM (μM)			SI
	*T. brucei*	RAW 264.7	
Compound **1**	7.3 ± 0.08	215.6 ± 0.9	29.5
Compound **2**	3.2 ± 0.09	292.1 ± 1.5	91.3
Compound **3**	4.5 ± 0.05	314.6 ± 1.9	69.9
DA	1.7 ± 0.07	138.7 ± 2.0	81.6

SI (selectivity index) was calculated as the ratio of the EC_50_ value in RAW 264.7 cell lines to that in *T. brucei*. DA = diminazene aceturate; SEM = standard error of the mean.

## Data Availability

Data is contained within the article or supplementary material.

## Supplementary Materials

The following are available online, Figure S1: GC-MS total ion chromatogram for compound **2**. Figure S2: Mass fragmentation and ATR-IR spectra for compound **2**. Figure S3: GC-MS total ion chromatogram for compound **3**. Figure S4: Mass fragmentation and ATR-IR spectra for compound **3**. Figure S5: UV–VIS spectra for compounds **2** and **3**. Figure S6: ^1^H and ^13^C NMR spectra for compound **2** in C_2_D_6_OS. Figure S7: ^1^H and ^13^C NMR spectra for compound **3** in CDCl_3_. Figure S8: Dose–response curves of compounds in *T. brucei*. Table S1: 1D and 2D NMR data for compound **2** in C_2_D_6_OS. Table S2: 1D and 2D NMR data for compound **3** in CDCl_3_. Table S3: Effect of compounds on ratio of nuclei and kinetoplasts in *T. brucei.*

## Sample Availability

Samples of compounds 1, 2 and 3 are available from the authors.

## Abbreviations

AT: African trypanosomiasis; *T. brucei*: *Trypanosoma brucei*; *Z. zanthoxyloides*: *Zanthoxylum zanthoxyloides* (Lam.) Zepern and Timler.
