# Supplementary material for: Chemical Derivatization and Characterization of Novel Antitrypanosomals for African Trypanosomiasis
Source: Molecules. 2021 Jul 25;26(15):4488. doi: 10.3390/molecules26154488 (PMC8347361; doi:10.3390/molecules26154488)
Supplement: Supplementary file 1 [file molecules-26-04488-s001.zip › molecules-1289963-supplementary.pdf]

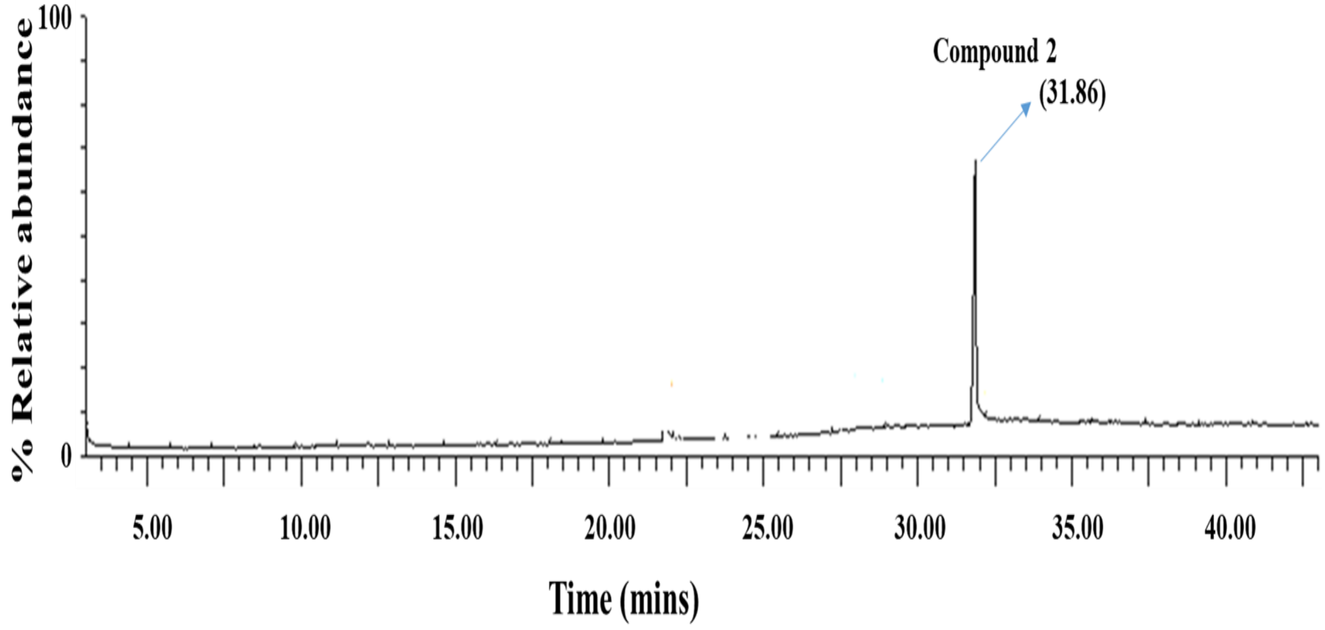

Compound **2** was eluted at a retention time of 31.86 mins

Figure S1: GC-MS total ion chromatogram for compound 2.

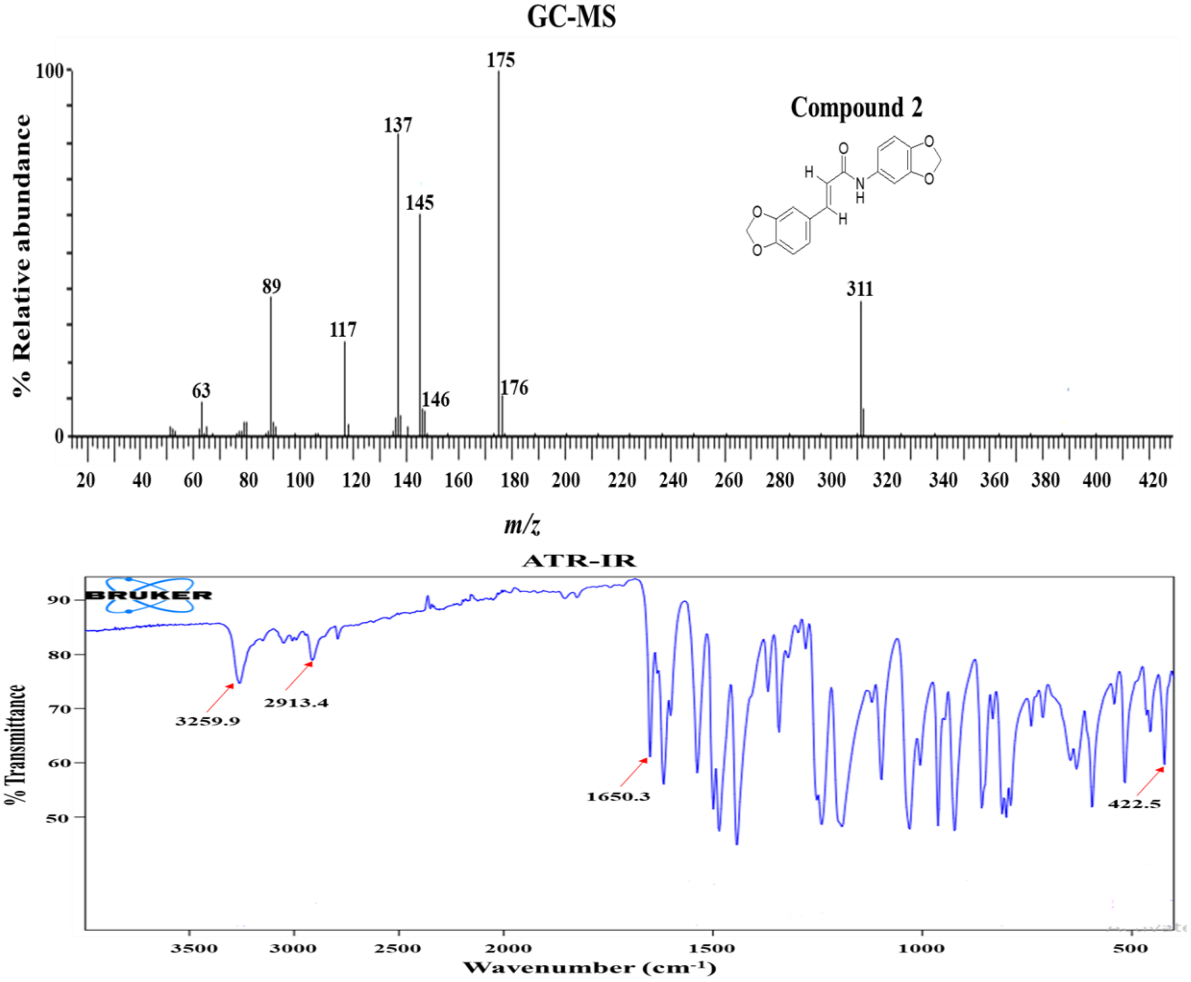

Mass fragmentation pattern of compound **2** indicated approximate *m/z* of 311. The IR spectra displayed absorbance intensities of N-H (1650-1580 cm^{-1}), C-C (1500-1400 cm^{-1}) and C-N (1250-1020 cm^{-1}) functionalities suggestive of aromatic and amide groups in compound **2**

Figure S2: Mass fragmentation and ATR-IR spectra for compound 2.

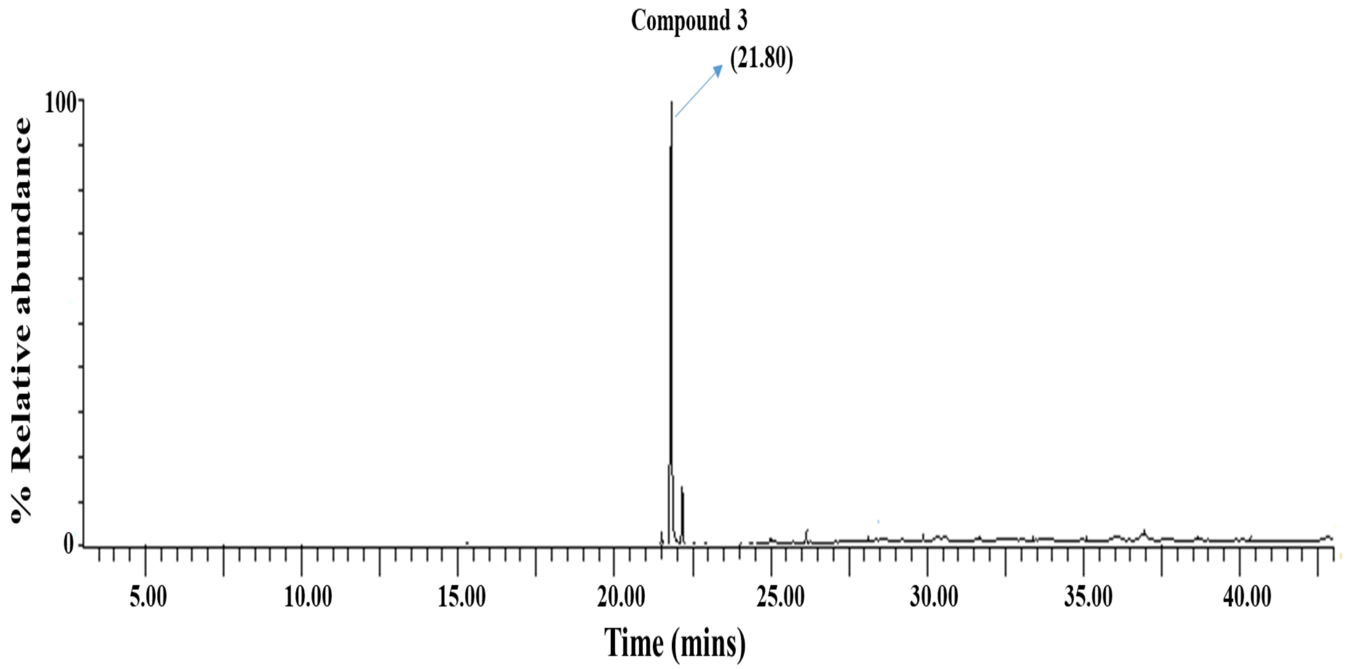

Compound **3** was eluted at a retention time of 21.80 mins

Figure S3: GC-MS total ion chromatogram for compound 3.

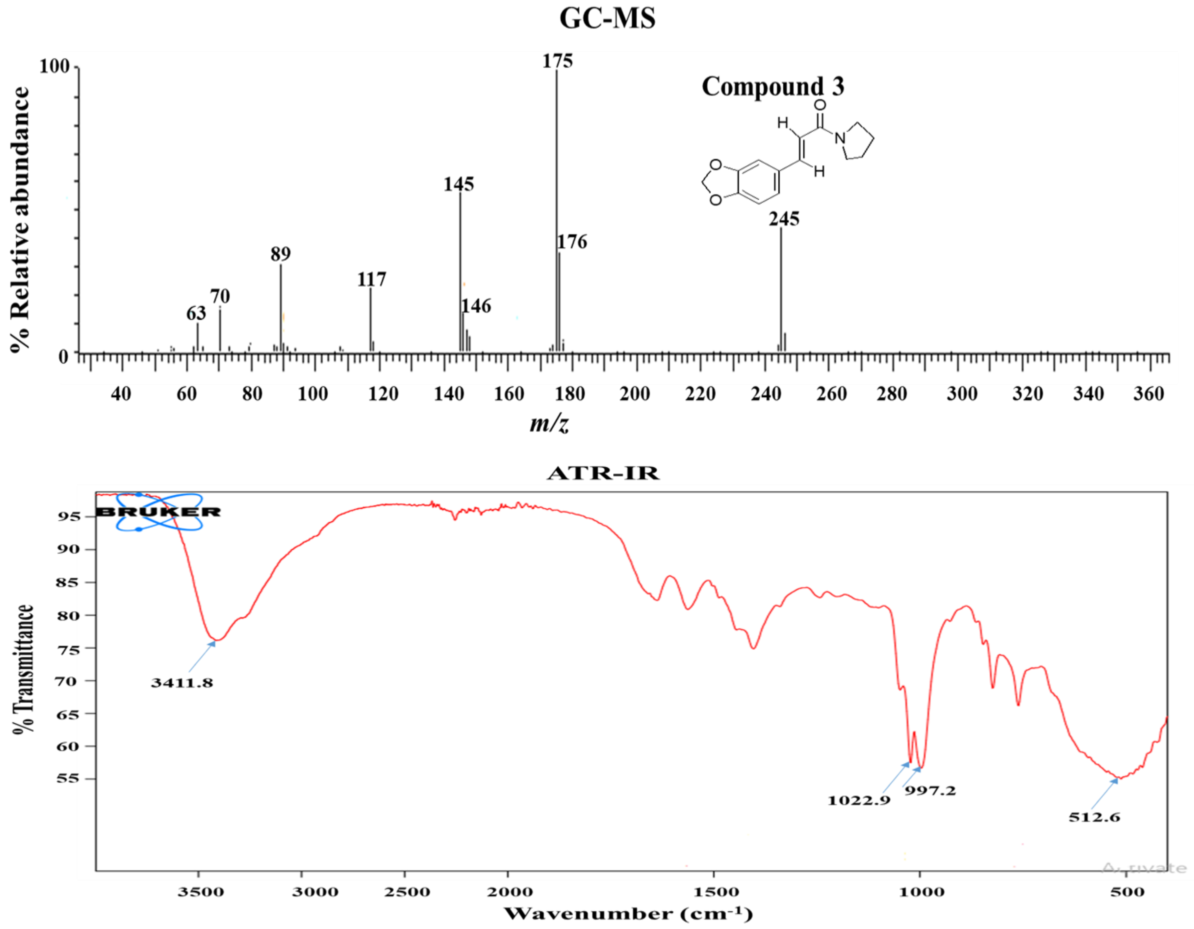

Mass fragmentation pattern of compound **3** indicated approximate *m*/*z* of 245. The IR spectra displayed absorbance intensities suggestive of C-C (1500-1400 cm^{-1}) and C-N (1250-1020 cm^{-1}) functionalities.

Figure S4: Mass fragmentation and ATR-IR spectra for compound 3.

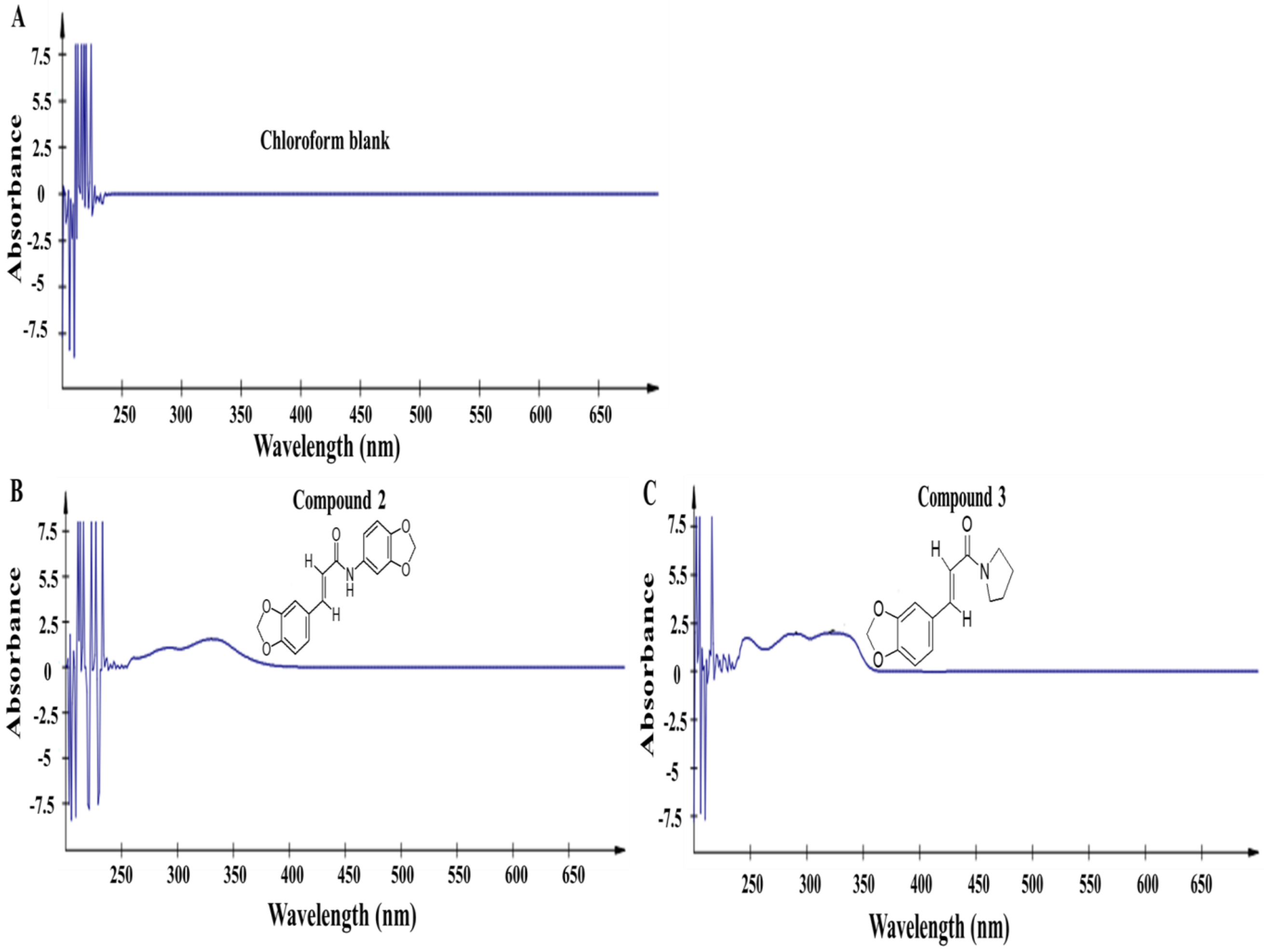

UV-VIS spectra were restricted to absorption bands within 250-350 nm. Chloroform was used as the blank solvent.

Figure S5: UV-VIS spectra for compounds 2 and 3.

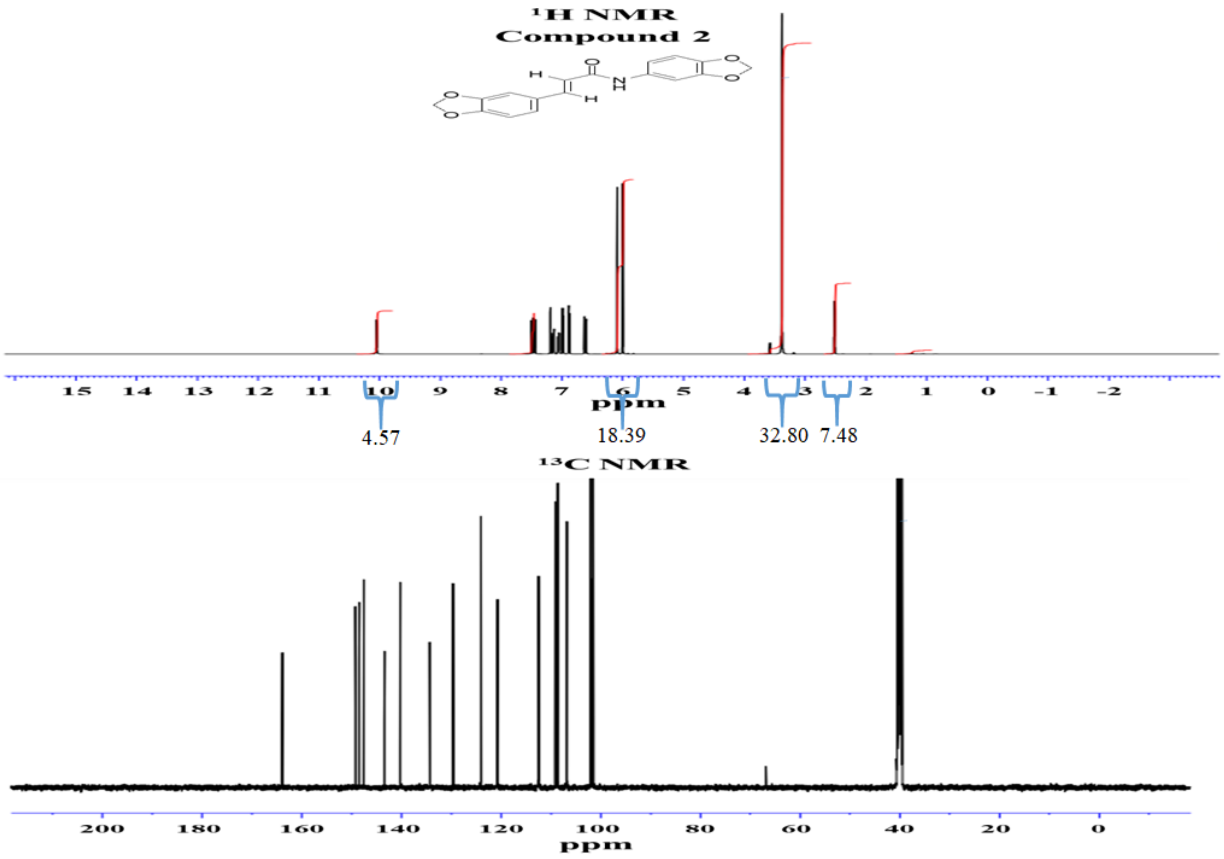

Figure S6: ^{1}H and ^{13}C NMR spectra for compound 2 in C_2D_6OS.

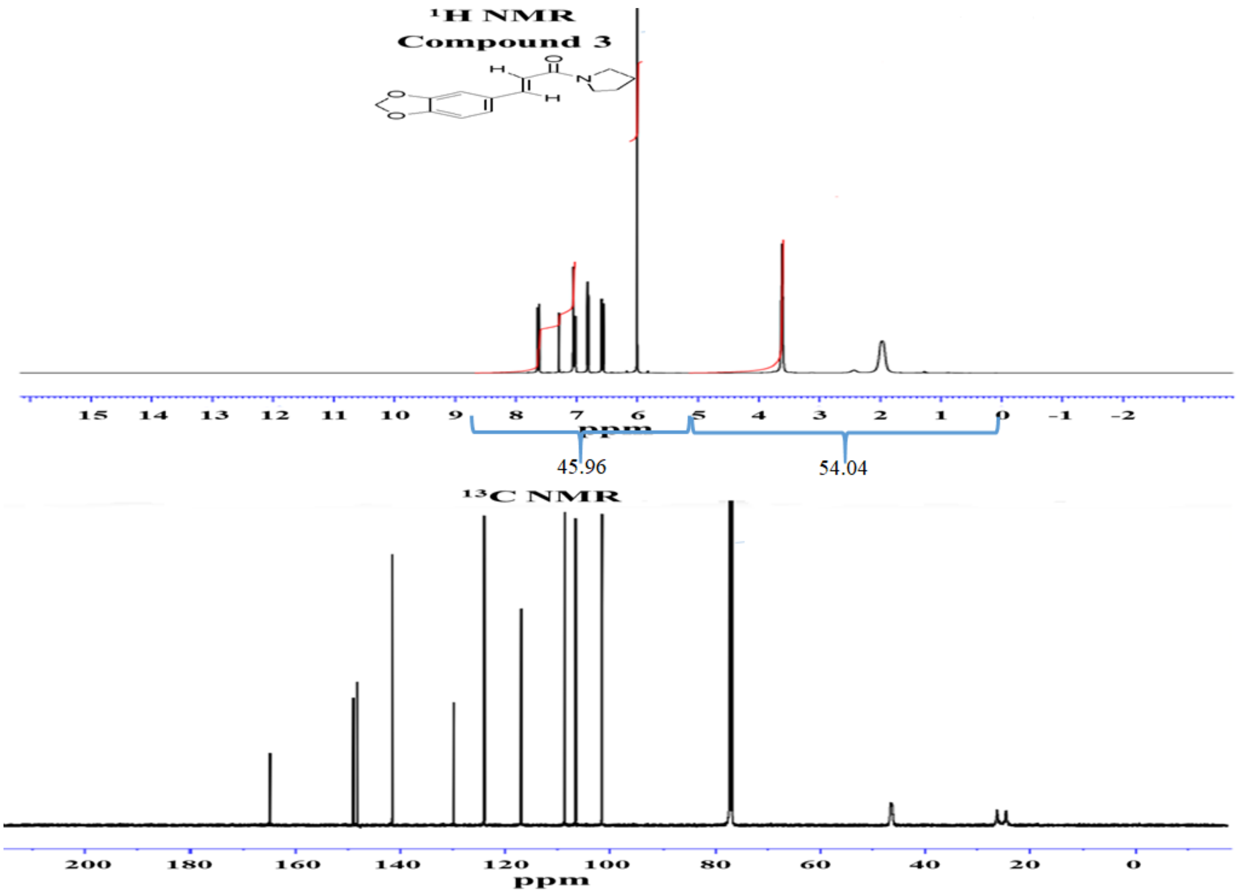

Figure S7: ¹H and ¹³C NMR spectra for compound 3 in $CDCl_3$.

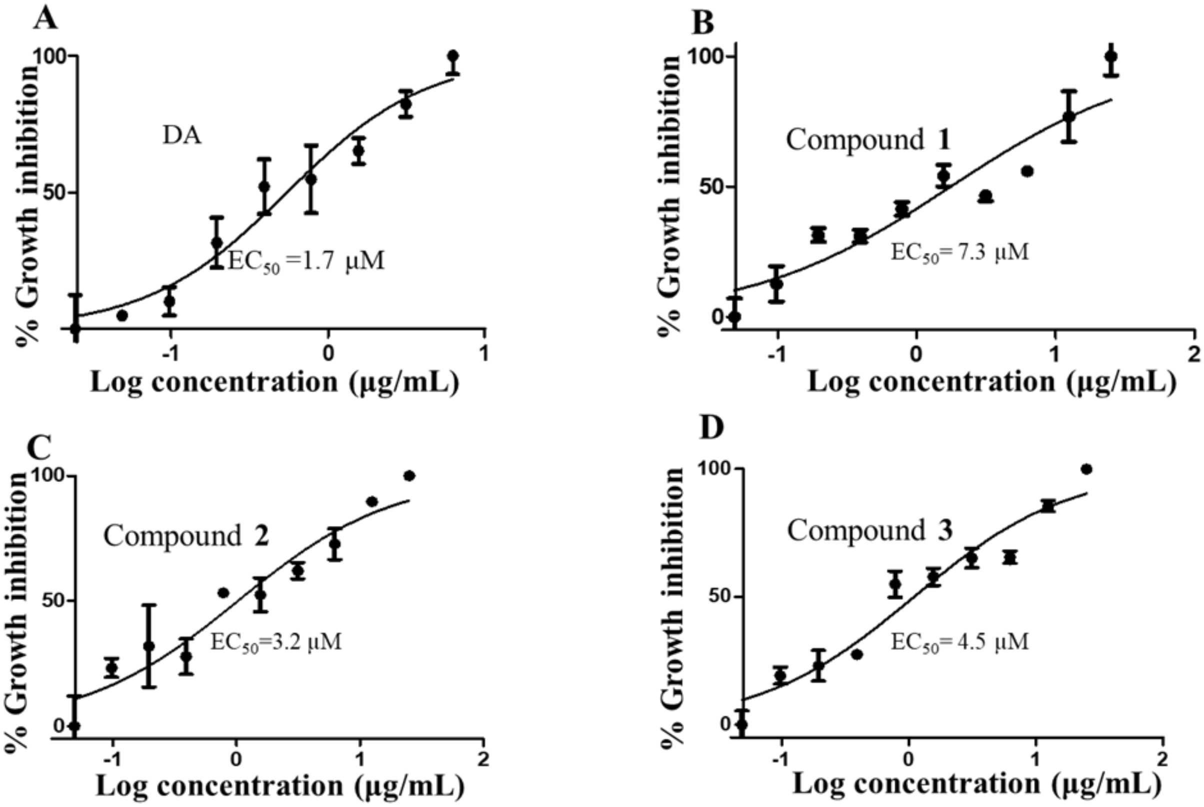

Dose-response curves and half-maximal effective concentration (EC_{50}) values for DA (A), compound **1** (B), compound **2** (C) and compound **3** (D) were modeled from quadruplicates in a non-linear regression using the Hill function. DA= diminazene aceturate.

Figure S8: Dose-response curves of compounds in *T. brucei*.

Table S1: 1D and 2D NMR data for compound 2 in C_2D_6OS.

Serial No	^{13}C	DEPT 135	HSQC	COSY	HMBC
1	66.8	CH_2	3.58	-	-
2	101.4	CH2	6	-	14,15
3	101.8	CH/CH_3	7.44	13	8,12*,14,15*
4	102	CH2	6.1	-	16,10,13
5	106.8	CH/CH3	7.19	-	17
6	108.6	CH/CH3	6.88	-	4*,8,12,14,15
7	109.1	CH/CH3	6.98	10	10,11,16
8	112.4	CH/CH3	7.05	-	3,6*,14
9	120.7	CH/CH3	6.63/6.60	13	11,18
10	124	CH/CH3	7.14	7	5,13,17
11	129.6	Cq	-	-	-
12	134.3	Cq	-	-	-
13	140.2	CH/CH3	7.5/7.48	3,9	5,9*,10,11*,18
14	143.4	Cq	-	-	-
15	147.5	Cq	-	-	-
16	148.5	Cq	-	-	-
17	149.2	Cq	-	-	-
18	163.9	Cq	-	-	-

DEPT = Distortionless Enhancement by Polarisation Transfer; HSQC = Heteronuclear Single Quantum Coherence; COSY = Correlation Spectroscopy; HMBC = Heteronuclear Multiple Bond Correlation; Cq = quaternary carbon; * = weak.

Table S2: 1D and 2D NMR data for compound 3 in $CDCl_3$.

Serial No	^{13}C	DEPT 135	HSQC	COSY	HMBC
1	24.4	CH2	1.96	3	-
2	26.1	CH2	1.97	3	-
3	46.2	CH2	3.61	1,2,7	1,2,13
4	101.4	CH2	6	6*	11,12
5	106.4	CH/CH3	7.05	-	8,10
6	108.5	CH/CH3	6.81	4*,7*,8	5,9,11
7	116.8	CH/CH3	6.55, 6.59	10,3*,6*	9,10,13
8	123.9	CH/CH3	7.02	6	6*,12
9	129.8	Cq	-	-	-
10	141.5	CH/CH3	7.628,6.581	7	5,7,8,9*,13
11	148.2	Cq	-	-	-
12	149	CH/CH3	7.02	6	5,10
13	164.9	Cq	-	-	-

DEPT = Distortionless Enhancement by Polarisation Transfer; HSQC = Heteronuclear Single Quantum Coherence; COSY = Correlation; HMBC = Heteronuclear Multiple Bond Correlation; Spectroscopy; Cq = quaternary carbon; * = weak.

Table S3: Effect of compounds on ratio of nuclei and kinetoplasts in *T. brucei*.

	Percentage cell population					
**Compounds**	**1N1K**	**1N2K**	**2N2K**	**1N0K**	**0N**	**>2N**
NC	70.09	22.43	4.21	2.80	0	0.47
DA	9.95	1.00	16.42	70.15	0	2.49
Compound **1**	53.88	37.50	5.17	3.02	0.43	0
Compound **2**	68.84	23.19	3.99	3.99	0	0
Compound **3**	69.40	26.12	1.12	3.36	0	0

DA = Diminazene aceturate; NC = Negative control; K = kinetoplast, N = nucleus.
